# *Aspergillus tubingensis:* A Rare Fungal Pathogen Complicating COVID‐19 Case

**DOI:** 10.1155/crdi/5831166

**Published:** 2025-12-11

**Authors:** Aiah M. Khateb, Fadwa S. Alofi, Abdullah Z. Almutairi, Mohammad A. Turkistani, Ziab Z. Alahmadey, Hessa A. Al-Sharif, Esam I. Azhar

**Affiliations:** ^1^ Department of Clinical Laboratory Sciences, Collage of Applied Medical Science, Taibah University, P.O. Box 344, Madina, 42353, Saudi Arabia, taibahu.edu.sa; ^2^ Health and Life Research Center, Taibah University, Madina, Saudi Arabia, taibahu.edu.sa; ^3^ Special Infectious Agents Unit-BSL3, King Fahd Medical Research Center, King Abdulaziz University, Jeddah, 21362, Saudi Arabia, kau.edu.sa; ^4^ Department of Infectious Diseases Department, King Fahad Hospital, P.O. Box 3177, Almadinah Almunawwarah, Saudi Arabia, kfmc.med.sa; ^5^ Department of Microbiology Laboratory, King Fahad Hospital, P.O. Box 3177, Almadinah Almunawwarah, Saudi Arabia, kfmc.med.sa; ^6^ Department of Clinical Laboratory, Ohud Hospital, Ministry of Health, Madinah, Saudi Arabia, moh.gov.sa; ^7^ Medical Laboratory Sciences Department, Faculty of Applied Medical Sciences, King Abdulaziz University, Jeddah, Saudi Arabia, kau.edu.sa

**Keywords:** *Aspergillus tubingensis*, case report, COVID-19, infections, resistance

## Abstract

**Background:**

Coronavirus disease 2019 (COVID‐19) has been associated with invasive fungal infection. Several COVID‐19 cases were complicated due to coinfection with *Aspergillus, Rhizopus, and Mucor* species. We present COVID‐19 with *Aspergillus tubingensis* coinfection.

**Case Presentation:**

A male patient presented with fever, cough, severe shortness of breath, and abdominal pain that persisted for a week. The patient was admitted to the ICU. The patient was diabetic and hypertensive, and COVID‐19 pneumonia was confirmed. The patient became septic, and his blood culture was positive for *Candida albican*s. His sputum culture was positive for *Acinetobacter* spp. The patient was treated with a broad range of antibiotics and antifungal treatment. His case was complicated by hospital‐acquired pneumonia; sputum culture was positive for *Aspergillus species.* Immediately, he developed septic shock, acute kidney injury, and disseminated intravascular coagulation (DIC). Postmortem molecular identification via ITS sequencing confirmed the presence of *Aspergillus tubingensis*.

**Conclusions:**

Invasive fungal infections are characterized by high mortality. Early diagnosis to the species level is essential for successful treatment.

## 1. Introduction

Invasive pulmonary aspergillosis (IPA) is an acute fungal infection with rising incidence and a high mortality rate. This increase is mainly due to an increase in patients with underlying diseases and immunosuppressed diseases, therapies, or new cryptic species [1]. Respiratory viral infections, especially influenza and coronavirus disease 2019 (COVID‐19), have been described as a risk factor for IPA coinfection [2, 3], particularly in cases of severe pulmonary abnormalities and acute respiratory distress syndrome (ARDS) [4]. The World Health Organization (WHO) reported 827,488 confirmed cases of COVID‐19, with 9549 deaths, and a total of 67,577,413 vaccine doses have been administered in Saudi Arabia between January 2020 and January 2023 [5]. Reports of COVID‐19 fungal coinfections were mainly caused by *Aspergillus, Rhizopus, Mucor*, and other mold species. Numerous studies have documented superinfections in COVID‐19 patients, with COVID‐19‐associated pulmonary aspergillosis (CAPA) frequently being reported. CAPA was further associated with risk factors such as age, prolonged stay in intensive care units (> 14 days), use of mechanical ventilators, ARDS, and comorbidities such as diabetes and obesity [6]. Diagnosis is challenging due to the nonspecific nature of symptoms. The well‐established criteria of the European Organization for Research and Treatment of Cancer/Mycoses Study Group (EORTC/MSG) may be applied to uncomplicated CAPA cases [2]. However, in the ICU setting, these methods are often limited and cannot be applied because of the patients’ severely compromised state. An enhanced criterion for ICU patients was created based on clinical signs and symptoms compatible with IPA, abnormal chest imaging of the lungs, and microbiological evidence of the presence of *Aspergillus* [3]. Failure or delay in the administration of the right drug contributes to increased mortality. In addition, azole‐resistant isolates potentially increase mortality to 50%–100%. CAPA causative agents included *Aspergillus fumigatus A. terreus, A. nidulans, and A. niger* [7]. Here, we describe the rare case of *Aspergillus tubingensis* in a SARS‐CoV‐2‐positive patient admitted to the ICU.

## 2. Case Presentation

**Figure 1:**
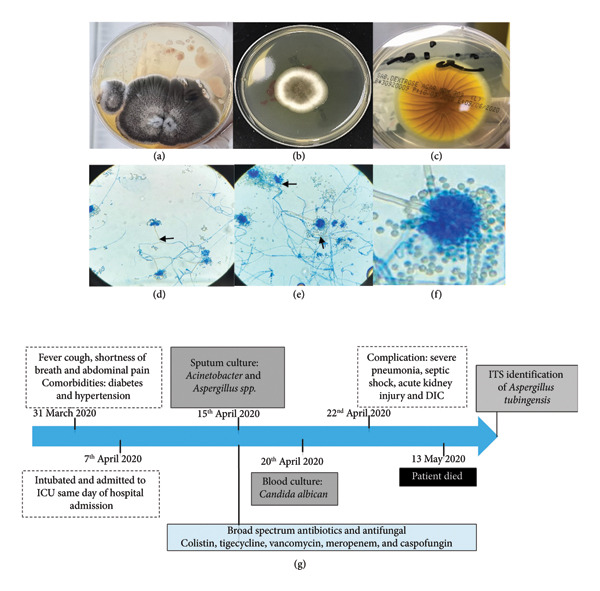
Morphological identification of *Aspergillus* species in a case of fatal polymicrobial coinfection. (a–c) Macroscopic examination of the respiratory culture on Sabouraud Dextrose Agar shows the growth of an *Aspergillus* species. (d–f) Microscopic examination reveals short, rough stalks arising from foot cells. The vesicle is round with phialides covering two‐thirds, bearing a double series of sterigmata. Conidia are rough, round (3.5 μm), and greenish‐brown to dark yellow‐brown. (g) A clinical timeline illustrating the rapid decline of a patient with diabetes and hypertension due to polymicrobial coinfection with *Acinetobacter*, *Aspergillus* tubingensis, and *Candida albicans*, leading to severe complications and death despite broad‐spectrum treatment.

A male patient in his seventies presented with fever, dry cough, severe shortness of breath, and abdominal pain that persisted for a week. The patient was admitted to the hospital on the 7th of April 2020. On the same day, he was intubated and admitted to the ICU, and a PCR of the nasopharyngeal swab confirmed that he was COVID‐19 positive. The patient’s medical history indicated that he was suffering from diabetes and high blood pressure. Other laboratory findings included complete blood count with low leucocytes and RBC count and severe neutropenia (WBC 0.817 [4–11 × 10^6^ KIU/L], RBC 2.996 [4‐5 × 10^6^ cell/µL], neutrophils 0.404 [2–7.5 × 10^3^ cells/µL], lymphocytes 0.130 [1–5.2 × 10^3^ cells/µL], and hemoglobin 8.01 [12–16 q/dL]).The patient also had high liver enzymes (alkaline phosphatase [ALP] 123 [31–91 SEC], aspartate aminotransferase [AST] 58 [15–41 U/L], alanine transaminase [ALT] 42 [1–37 U/L], total bilirubin 303.30 [5–13 μmol/L], high direct bilirubin 170.10 [0–3.07 μmol/L], and low albumin 23.90 [35–50 μmol/L]) Table S1. His sputum culture was positive for *Acinetobacter* spp., and a fungal culture was performed. *Aspergillus species* recovered from sputum culture were then purely isolated for examination. The isolated fungal colony, obtained from the original clinical sample, was cultured on Sabouraud Dextrose Agar and incubated in a standard microbiological incubator at a temperature of 25°C–30°C (Forma Scientific Incubator, Germany) for 7 days. Macroscopic examination of the mature colony revealed a flat, velvety, and powdery texture with a yellowish‐brown to light‐brown coloration, and a similar pigmentation on the reverse side (Figures 1(a), 1(b), and 1(c)). For definitive species confirmation, a microscopic mount was prepared using lactophenol cotton blue stain. Microscopic analysis revealed short, rough‐walled conidiophores terminating in globose vesicles, from which a biseriate arrangement of phialides produced the globose, finely roughened conidia. No Hülle cells were seen (Figures 1(d), 1(e), and 1(f)). Later, for definitive identification of *Aspergillus species*, the sample was sent for molecular identification. The patient’s hospital course was complicated by hospital‐acquired pneumonia and ARDS, which were confirmed. In the interim, the patient was treated with a broad range of antibiotics and antifungals, which included colistin, tigecycline, vancomycin, meropenem, and caspofungin. Soon after, the patient became septic, and his blood culture grew *Candida albicans* (Figure 1(g)). The samples were cultured aerobically and anaerobically using the fully automated BacT/Alert and BACTEC‐fx Microbial Detection System until a signal‐positive alarm was sounded or for a maximum of 5 days (bioMérieux, Durham, NC, USA). BACTEC Myco/F lytic culture vials for fungi were used for high‐risk cases. Positive blood culture bottles were processed for further identification using the VITEK 2 system (bioMérieux, Marcy‐L’Étoile, France) for identification and sensitivity according to the manufacturer’s instructions. Antifungal susceptibility reporting criteria were interpreted based on the updated guidelines of the CLSI (resistance, sensitivity, and intermediate resistance). Immediately, the patient’s case was complicated due to septic shock, acute kidney injury, and disseminated intravascular coagulation (DIC). Sadly, the patient died, and an autopsy was not performed.

## 3. Molecular Identification

**Figure 2:**
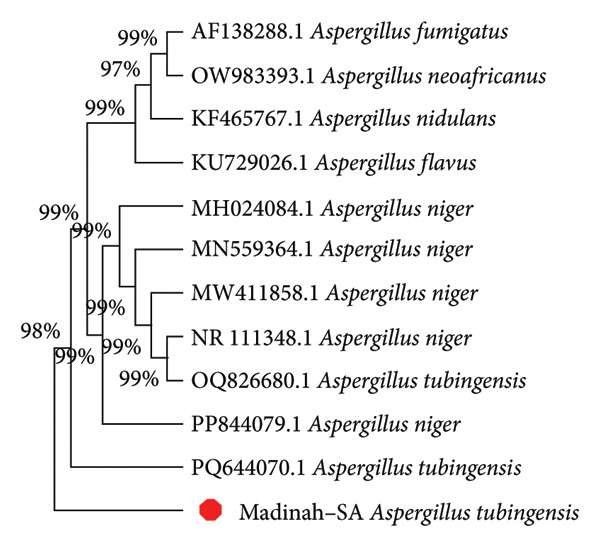
Phylogenetic analysis of a clinically or environmentally relevant *Aspergillus tubingensis* isolate from Madinah, Saudi Arabia. The unrooted phylogenetic tree, constructed using the neighbor‐joining method based on ITS gene sequencing data, shows the genetic relationships between the Madinah isolate and other reference strains of *Aspergillus* species. The bootstrap values at the nodes indicate the percentage of replicate trees that support the branching pattern’s topology. The Madinah isolate (indicated by the red dot) clusters directly with a reference strain of *A. tubingensis*, confirming its species identification. This clade, in turn, is nested within a larger, highly supported clade containing several *A. niger* strains, demonstrating the close evolutionary relationship between the two species within the *Aspergillus* section *Nigri*.

After the patient died, molecular identification (ITS sequencing) confirmed *Aspergillus tubingensis*. The molecular identification was performed at the Special Infectious Agents Unit‐BSL3, King Fahd Medical Research Center, King Abdulaziz University, Jeddah, Saudi Arabia. Fungal DNA was extracted from cultured spores using a PBS–Tween 20 and glass bead lysis protocol [8], followed by purification of the supernatant with the ZR Fungal/Bacterial DNA MiniPrep Kit (Zymo Research, USA). The ITS region used for detection of fungal DNA was performed by amplifying with ITS1/ITS4 primers [9] using DreamTaq Green Master Mix (Thermo Fisher Scientific Inc, USA). The amplicons (∼600 bp) were gel‐purified using Norgen DNA Gel Extraction Kit (Norgen Biotek Corp) according to the manufacturer’s instructions and subsequently subjected to bidirectional Sanger sequencing analysis using an ABI Prism Big Dye Terminator Cycle Sequencing Kit v3.1 on an ABI 3500 Genetic Analyzer (Applied Biosystems, USA). The fungi sequence result was aligned using BLAST in GenBank and fungidb.org for identification and for subsequent phylogenetic analysis Figure 2. The phylogenetic analysis, based on the ITS fungal gene region, confirmed the identity of the Madinah–SA isolate as *Aspergillus tubingensis*. The analysis showed that the Madinah–SA strain is most closely related to another *A. tubingensis* isolate strain (labeled PQ644070.1), with a shared lineage forming a strongly supported clade as shown by their tight clustering on the tree. This clade, in turn, is nested within a larger group containing multiple *A. niger* strains, a relationship supported by high bootstrap values of 99%. This is highly relevant because *A. tubingensis* is a cryptic species within the *A. niger* complex, meaning they are morphologically similar but genetically distinct. This demonstrates a clear genetic relatedness between the Madinah–SA isolate and other reference strains of *A. tubingensis* and its sister species, *A. niger*.

## 4. Discussion

We report a rare case of CAPA in a 72‐year‐old, neutropenic, diabetic, and hypertensive patient. The classification of *Aspergillus tubingensis* as a rare pathogen is based on its low reported incidence and specific host association. Infections from this fungus are primarily documented in isolated case reports and small case series, almost exclusively in severely immunosuppressed patients. Its rarity is also partly due to diagnostic challenges, as the fungus has historically been misidentified as other morphologically similar species, such as *A. niger* or *A. ustus*, and requires advanced molecular sequencing for accurate differentiation.

As an environmental strain commonly found in soil and indoor environments, *A. tubingensis* belongs to the *Aspergillus* section *Nigri*, which was the second most common fungal group found in indoor homes in Madinah City, Saudi Arabia [10, 11]. In humans, it has been reported to cause a variety of infections, including onychomycosis, endocarditis, and respiratory infections [12–14].

Consistent with our findings, a case report from Iran by Khodavaisy et al. documented proven pulmonary aspergillosis caused by *A. tubingensis* in a 59‐year‐old COVID‐19 patient [14]. This case highlights the importance of early diagnosis and management of invasive fungal infections (IFIs) in critically ill COVID‐19 patients, as they are at an increased risk for such coinfections. In another case of respiratory infection. Hase et al. reported *A. tubingensis* as the cause of bronchopulmonary oxalosis in a non‐neutropenic patient [15]. Despite the high mortality rate associated with this condition and the known azole resistance of this species in Japan, the patient was successfully treated with voriconazole monotherapy, suggesting the importance of detailed susceptibility testing to guide treatment [15]. In a broader context, a case series by Fernandez‐Pittol et al. examined the frequency of cryptic *Aspergillus* species in a hospital setting and identified one isolate of *A. tubingensis* among a total of ten cryptic species [1]. This study found that most patients with these rare infections were immunosuppressed, with a high mortality rate of 40%. A separate study by Gautier et al. found *A. tubingensis* to be the fifth most frequent mold isolated from clinical samples, primarily from patients with chronic respiratory conditions, suggesting its role in disease progression in respiratory infections [16].

Therapeutic management of *A. tubingensis* infections is challenging due to its complex drug susceptibility profile [17]. Research indicates that *A. tubingensis* isolates often exhibit high azole resistance relative to other *Aspergillus* species, with a study by Hashimoto et al. finding a very high rate of azole resistance (79.5%–89.7%) [17]. This resistance may be linked to the overexpression of the *cyp51A* gene. The lack of defined clinical breakpoints and epidemiological cutoff values for this species further complicates treatment [17]. Another case report with *A. tubingensis* also highlights that surgery is crucial for a positive outcome and that, in vitro, only amphotericin B had significant activity against the fungus’s biofilms, a key therapeutic challenge [14]. The poor clinical course for patients with *A. tubingensis* infection can therefore be as unfavorable as that of azole‐resistant *A. fumigatus* infections [9].

In China, a study by Yusufu et al. reported that *Aspergillus tubingensis* was a remarkably common clinical isolate in Xinjiang, China, ranking as the third most prevalent *Aspergillus* species [18]. This finding, derived from molecular diagnostics, underscores the need for advanced identification methods to accurately assess its true prevalence. While the study reported general susceptibility to itraconazole, it also documented a significant rate of azole nonsusceptibility linked to *cyp51A* gene mutations, reinforcing that resistance remains a serious concern [18]. These data, along with our case report, highlight the growing clinical relevance of *A. tubingensis* and the urgent need for precise diagnosis to guide therapy.

The phylogenetic results are highly relevant as they provide molecular confirmation of a specific *A. tubingensis* strain’s presence in Madinah, Saudi Arabia. The close genetic relationship between the *A. tubingensis* isolate and the *A. niger* reference strains is significant because *A. tubingensis* is a cryptic species within the *A. niger* complex [14]. This finding underscores the fact that these species, while morphologically similar, are genetically distinct, highlighting the critical importance of using advanced molecular methods, such as ITS sequencing, for accurate species‐level identification. Without such precision, this clinically relevant strain might be misidentified, potentially impacting treatment strategies and epidemiological understanding in the region [9].

Finally, molecular‐based resistance screening, even in the absence of a full culture and sensitivity, is paramount. Genetic markers associated with antifungal resistance, such as mutations in the *cyp51A* gene in *Aspergillus*, can be detected directly from a clinical sample [3]. Had these tests been available, the clinicians could have rapidly determined the likely resistance profile of the infecting organism and selected an alternative, effective antifungal agent, such as a liposomal amphotericin B or other, potentially altering the patient’s outcome.

The escalating global incidence of IFIs in critically ill COVID‐19 patients necessitates a shift toward comprehensive diagnostic vigilance that extends beyond the common *Aspergillus* species. While the multicentric mucormycosis study confirms that Mucorales are another major cause of lethal fungal complications, driven by host factors such as poor glycemic control and ICU admission [19]. a concerning case series by Patil et al. (2023) further broadens this microbial risk spectrum [20]. The latter documents fatal infections caused by rare and unconventional fungi such as *Neurospora*, *Cladosporium*, and *Fusarium*, which are typically considered nonpathogenic. These outcomes underscore that the unique immune dysregulation caused by COVID‐19 facilitates devastating infections by a diverse range of fungal agents. Consequently, effective management of COVID‐19‐associated IFIs requires broad vigilance and is critically dependent on early, species‐level identification paired with aggressive, multimodal therapy.

This case presented a stark reminder that while clinical and radiological findings are suggestive, they are often insufficient for guiding effective therapy in complex fungal–viral coinfections. The lack of molecular testing due to reagent shortages during the COVID‐19 crisis represents a significant and tragically illustrated gap in diagnostic capabilities, highlighting the need for robust and readily available molecular surveillance systems to combat these emerging and drug‐resistant pathogens. It also reinforces that environmental fungi, typically considered less pathogenic, can cause life‐threatening disease in severely compromised hosts. Ultimately, this case underscores the urgent need for rapid diagnostic and antifungal resistance screening methods to guide early, targeted therapy and improve patient prognosis, particularly in regions where less common *Aspergillus* species are endemic. Moving forward, comprehensive surveillance of hospital environments and the establishment of registries for these rare cases will be essential to inform clinical guidelines and mitigate the high mortality associated with these complex infections.

## 5. Conclusions

Patients with COVID‐19 and IPA coinfection have a poor prognosis. Registries and larger cohort studies are needed to understand geographical variation, diagnostic criteria, and the role of immunosuppressive therapy with COVID‐19 as a risk factor for IPA. Screening of hospital environmental surveillance is needed to evaluate the prevalence of IPA in patients admitted to the ICU.

**Nomenclature**
ALP: Alkaline phosphatase
ALT: Alanine transaminase
ARDS: Acute respiratory distress syndrome
AST: Aspartate aminotransferase
BMT: Bone marrow transplantation
CAPA: COVID‐19‐associated pulmonary aspergillosis
CLSI: Clinical and Laboratory Standards Institute
DIC: Disseminated intravascular coagulation
EORTC/MSG: European Organization for Research and Treatment of Cancer/Mycoses Study Group
ICU: Intensive care unit
IPA: Invasive pulmonary aspergillosis
RBC: Red blood cell
WBC: White blood cell
WHO: World Health Organization

## Ethics Statement

This patient’s sample was referred from Ohud Hospital for microbiology diagnosis at King Fahad Hospital in Medina (KFH), where it was diagnosed. The paper has been sufficiently anonymized not to cause harm to the patient’s family. No ethical approval number was generated.

## Consent

The head of the medical team physician’s agreement was obtained, and patient written informed consent was not obtained since the patient passed away and patient’s relatives could not be reached because all telephones were out of service, and there was no forwarding address to reach them. The head of the medical team of physicians has exhausted all means to reach them and has anonymized all the patients’ images and data to avoid hurting the patient or their family.

## Disclosure

All authors have read and agreed to the published version of the manuscript.

Part of the article was presented in conference the 10th Advances Against Aspergillosis & Mucormycosis Conference in 2022, aaam2022.org. The abstract supplement was not published in any journal.

## Conflicts of Interest

The authors declare no conflicts of interest.

## Author Contributions

Conceptualization and formal analysis: Aiah M. Khateb. Investigation: Fadwa S. Alofi, Mohammad A. Turkistani, Abdullah Z. Almutairi, and Ziab Z. Alahmadey. Molecular identification: Hessa A. Al‐Sharif, Esam I. Azhar, and Aiah M. Khateb. Resources, writing–original draft preparation, and editing: Aiah M. Khateb. Review–writing: Aiah M. Khateb, Fadwa S. Alofi, Ziab Z. Alahmadey, Abdullah Z. Almutairi, and Mohammad A. Turkistani.

## Funding

This research received no external funding.

## Supporting Information

Table S1. Patient laboratory findings and normal range.

## Supporting information


**Supporting Information** Additional supporting information can be found online in the Supporting Information section.

## Data Availability

The data that support the findings of this study are available in the supporting information of this article.
