# Supplementary material for: Aspergillus tubingensis: A Rare Fungal Pathogen Complicating COVID‐19 Case
Source: Case Rep Infect Dis. 2025 Dec 11;2025:5831166. doi: 10.1155/crdi/5831166 (PMC12714113; doi:10.1155/crdi/5831166)
Supplement: Supplementary file 1 — Supporting Information Additional supporting information can be found online in the Supporting Information section. [file CRDI-2025-5831166-s001.docx]

Supplementary data

Table S1. Patient laboratory findings and normal range

| Laboratory test | Result | Normal range | Comment |
| --- | --- | --- | --- |
| Complete blood count | | | |
| WBC | 0.817 | 4-11x10^6^ KIU/L | Low |
| RBC | 2.996 | (4-5 x10^6^ cell/µL), | Low |
| Neutrophils | 0.404 | (2-7.5 x 10^3^ cells/µl), | Low |
| Lymphocytes | 0.130 | (1-5.2 x10^3^ cells/µl) | Low |
| Hemoglobin | 8.01. | (12-16 q/dL) | Low |
| Liver function tests | | | |
| Alkaline phosphatase (ALP) | 123 | (31-91 SEC) | High |
| aspartate aminotransferase (AST) | 58 | (15-41 U/L) | High |
| Alanine transaminase (ALT) | 42 | (1-37 U/L) | High |
| Total bilirubin | 303.30 | (5-13 µmol /L) | High |
| Bilirubin | 170.10 | (0-3.07 µmol/L) | High |
| Albumin | 23.90 | (35-50 µmol/L). | Low |
